# Stress Priming in Reading and the Selective Modulation of Lexical and Sub-Lexical Pathways

**DOI:** 10.1371/journal.pone.0007219

**Published:** 2009-09-29

**Authors:** Lucia Colombo, Jason Zevin

**Affiliations:** 1 Dipartimento di Psicologia Generale, University of Padua, Padua, Italy; 2 Institute for Developmental Psychobiology, Weill-Cornell Medical College, New York, New York, United States of America; University of Granada, Spain

## Abstract

Four experiments employed a priming methodology to investigate different mechanisms of stress assignment and how they are modulated by lexical and sub-lexical mechanisms in reading aloud in Italian. Lexical stress is unpredictable in Italian, and requires lexical look-up. The most frequent stress pattern (Dominant) is on the penultimate syllable [*laVOro* (work)], while stress on the antepenultimate syllable [*MAcchina* (car)] is relatively less frequent (non-Dominant). Word and pseudoword naming responses primed by words with non-dominant stress – which require whole-word knowledge to be read correctly – were compared to those primed by nonwords. Percentage of errors to words and percentage of dominant stress responses to nonwords were measured. In Experiments 1 and 2 stress errors increased for non-dominant stress words primed by nonwords, as compared to when they were primed by words. The results could be attributed to greater activation of sub-lexical codes, and an associated tendency to assign the dominant stress pattern by default in the nonword prime condition. Alternatively, they may have been the consequence of prosodic priming, inducing more errors on trials in which the stress pattern of primes and targets was not congruent. The two interpretations were investigated in Experiments 3 and 4. The results overall suggested a limited role of the default metrical pattern in word pronunciation, and showed clear effect of prosodic priming, but only when the sub-lexical mechanism prevailed.

## Introduction

According to most current models, reading aloud a word or pseudoword involves a mechanism transcoding orthography to phonology. Whether this occurs by means of application of default rules in addition to lexical look-up, as in the dual route model [1], [2] or by probabilistic constraint satisfaction in mapping between distinct codes [3]–[5] the output of this mechanism is described as a phonological representation that by some unspecified process gets to the articulation stage, and is then spoken. Very few details have been provided on the processes intervening between the formation of an abstract phonological code and its transformation into an articulatory code (although models of speech production vary in their compatibility with either approach; compare [6] with [7], or [8]). In addition, most current studies have been carried out in English – a language with an inconsistent orthography to phonology system – mainly with monosyllables, and with very little attention to the way stress is assigned (but see [9], [10]). In contrast, models of production have recently attempted to detail the mechanisms and representations involved during phonological encoding, the factors that affect this process, including stress placement, and how it leads to articulation. The current study is an attempt to fill the gap between these two domains in the literature, by investigating stress assignment in reading aloud with a pathway priming procedure. We examined these effects in Italian, which affords a unique perspective on the issue because of the role of lexical stress in mappings from spelling to sound.

### Stress, “regularity” and spelling-to-sound correspondence in Italian

Italian is a language with regular spelling-sound correspondences at the segmental level, but unpredictable stress [11], [12]. While disyllabic words are stressed almost exclusively on the penultimate syllable, three-syllabic words are more variable. For about 70% of these words, stress is on the penultimate syllable, while for a smaller percentage (about 20%), stress is on the initial (antepenultimate) syllable. The bias toward penultimate stress results in a “regularity” advantage for penultimate words in reading aloud [11], [12], that interacts with frequency such that it is much smaller for high-frequency words. This advantage is apparent in shorter latencies to dominant-stress (penultimate syllable) words, and in the tendency to assign a dominant stress to novel words [11]. In addition to the overall bias toward penultimate stress, particular phonological and orthographic neighborhoods, called *stress neighborhoods* – defined by the vocalic nucleus of the penultimate syllable and the last syllable – have their own sub-regularities [11]–[14]. For example, the word *bam'bino* has dominant stress, as it is stressed on the penultimate syllable, and is consistent, because most words ending in -INO have dominant stress as well (see Table 1 for examples). Similarly, in the word ‘*tavolo* (table), the unit –OLO (i.e., the unit formed by the nucleus of the penultimate syllable plus the last syllable) defines a neighborhood in which most words take non-dominant (antepenultimate) stress and is therefore “irregular” but consistent [11]–[14]. Interestingly, despite their “irregular” stress pattern, these items are sometimes named more rapidly than items such as *indi'ano* (Indian), which has many “irregular” neighbors, but is itself regular [14]. A similar advantage for irregular consistent words has been found in English for monosyllabic words [15], [16]. Consistency effects have also been found in nonword pronunciation: although nonwords are likely to be assigned a dominant stress pattern, this bias can be modulated by “stress neighborhood,” that is, the percentage of words sharing the stress pattern and the segments contained in the nucleus of the penultimate syllable plus the last syllable (i.e., all the words ending in –OLO, like ‘*tavolo* and sharing the same stress pattern from a consistent stress neighborhood; [11]).

**Table 1:** Examples of Italian words with dominant and non-dominant stress pattern and a consistent or inconsistent neighborhood.

	Dominant stress (Penultimate syllable)	Non-dominant stress (Initial syllable)
**Consistent**	ge LA to (ice cream)	SCA po lo (bachelor)
**Inconsistent**	in DIA no (Indian)	MAC chi na (machine)

In order to pronounce a word correctly, the representation computed in the naming process must be congruent with the corresponding *lexical* representation; stress placement in Italian is unpredictable, however, even considering the bias toward dominant stress and neighborhood characteristics. Taking these and other aspects of Italian spelling into account, Colombo [11], [12] proposed that three different mechanisms may be involved in the process of stress assignment in Italian, and must be considered in its modeling. One mechanism can take as input the sequence of phonemes derived from the orthography-to phonology mapping, and put it in correspondence with an independently computed metrical pattern. Such a mechanism would be subject to the overwhelming dominance of penultimate syllable stress in Italian, and would be biased to apply it to the phoneme sequence. The result is a stressed phonological pattern that may or may not correspond to the correct word in the lexicon. In this sense it can be considered a non-lexical mechanism. This notion is similar to a default mechanism based on the regularities of a language suggested by other authors [7], [17], [18]. There is also anecdotal evidence for this mechanism. For example, foreigners who try to pronounce words they do not know tend to stress Italian words on the penultimate syllable. Moreover, experimental evidence from stress assignment on nonwords suggests that Italian speakers too are more likely to stress new words on the penultimate syllable [11]–[13], [19] The data for real, known words are less clear.

In addition to a mechanism that applies the default stress in all instances, Colombo [11] proposed a sub-lexical mechanism that takes as input the syllabified representation of a word (or pseudoword) and assigns stress on the basis of neighborhood consistency (or inconsistency). For example, if the final two syllables belong to a dominant stress neighborhood, the phonetic characteristics of its segments, in particular the vowel (nucleus) of the penultimate syllable – its duration and intensity – will be consistent with a stressed syllable. Evidence in favor of the effects of neighborhood consistency has been found in a number of studies of novel and familiar words with normal adults and patients with dementia of the Alzheimer type [11]–[14].

Finally, in order to explain the fact that, despite the inconsistencies of the spelling-to-stress mapping, words are mostly pronounced with the correct stress, there must be a mechanism that matches the resulting phonetic sequence with a learned pronunciation specific to a given word. This notion is congruent with data showing that stress dominance only affects low frequency words [11] suggesting slower access to the pronunciation (and stress placement) of low, relative to high frequency words. Data from Greek [18], which is similar to Italian with respect to stress assignment, are also consistent with this model: Greek readers also make use of both lexical information, and a default metrical pattern in assigning stress.

Although for the sake of simplicity we discuss our data in terms of a dual-route model, we should point out that the phenomena described here can also be explained in terms of connectionist models, which are sensitive to both the overall statistics of the input (in this case, the dominant stress pattern) and more specific statistics at different grain sizes [4], [20]. For example, Harm & Seidenberg's [5] model of English reading contains a set of connections that maps directly from spelling to sound, which by itself generates effects of both regularity – items in which individual graphemes are assigned a less probable pronunciation, e.g., I pronounced as /aI/ in PINT – and effects of body-level consistency – e.g., O pronounced as /a/ in DOLL, despite overlap with ROLL, TOLL and POLL [4], [15], [16], [20]. An analogous mechanism might be at work in stress assignment for Italian. In fact, a model of stress assignment developed by Zevin & Joanisse 2000, unpublished manuscript) successfully simulated the influence of precisely the kind of stress neighborhood identified in Italian on pronunciation of nonwords by English speakers. In order to simulate the experiments reported here, such a model would need to be extended so that it included both direct and semantically-mediated mappings from spelling to sound, and would further require some mechanism for selectively controlling the relative dependence on these pathways, and on contextual influences.

### Stress in speech production

Stress assignment is part of the process of word form encoding in word production, during which segmental and metrical information is retrieved. The most influential theory in the field [7], [21] states that during phonological encoding the metrical pattern of a word, consisting of the number of syllables and the location of main stress, is computed separately from segmental information, resulting in a metrical frame into which the phonemic segments are inserted. The resulting syllables are used as pointers to retrieve articulatory/phonetic plans from a syllable store (*syllabary*). In this framework, a default rule is applied to most of the words, while for a small percentage of words the stress pattern is retrieved from the lexicon.

The model of stress assignment advanced by Levelt and collaborators [7] was developed in the context of Dutch, but is partially congruent with some aspects of the model proposed by Colombo [11], [12] for Italian. For example, the idea that a default is applied, reflecting the predominance of a stress pattern is similar in the two models, (although in [7], it is described as a rule mechanism, while in Colombo it is described in terms of a rhythmic pattern implicitly learned by speakers).

It is important to note the retrieval of the stored phonological form of a word in the lexicon is an operation that is somewhat task dependent. For example, it is required in metrical encoding in a picture naming task, in which the input is access to semantics and from here to the phonological output lexicon. However, in word reading it is not necessary to postulate that the output of the orthography –to-phonology mechanism has obligatory contact with the lexicon, or that generating a pronunciation is a consequence of the retrieval from the lexicon. In a dual route framework, the phonological representation of a word can be derived from a sub-lexical mechanism. Nonword reading, for example, clearly does not involve retrieval of a pronunciation from the lexicon. Therefore, when we model the process of speech production from reading aloud, we have to assume that the phonological representation that is formed during phonological encoding is not necessarily derived from a memory store, and can be non-lexical.

There is empirical evidence supporting this claim. Miceli & Caramazza [22] described a patient, CLB, who showed relative sparing of the ability to read words and nonwords, while he was impaired in the oral and written production of words. His reading performance was best described in terms of impaired access to lexical phonological representations, with a spared orthography-to-phonology conversion mechanism, that allowed him to read words and nonwords correctly at the segmental level (given the regularity of Italian at this level). Critically, he produced many stress errors in a manner consistent with sub-lexical assignment of stress. He made significantly more stress errors when the syllabic structure of the word did not require a specific stress pattern, but was lexically determined. Miceli & Caramazza inferred from the patient's data the existence of a non -lexical mechanism for assigning stress, and that the output of the orthography-to-phonology conversion mechanism is a phonological representation that is syllabically specified. The implication would be that stress can be applied both lexically and non lexically.

An interesting aspect to note was that, although CBL showed a tendency to produce more stress errors on non-dominant stress words than on dominant stress words, the difference was not significant. That is, the patient did not show a significant stress effect, with an advantage for dominant stress words, as would be expected assuming that the lexical mechanism was impaired, and dominant stress was applied by default. In contrast, he did show a significant tendency to assign dominant stress to nonwords. One possibility to explain this pattern of data would be to assume that there is no default application of dominant stress. This view is congruent with an interpretation of results by Burani and Arduino [14], who argued that their data from normal readers supported only the existence of stress neighborhood effects, not of a stress effect reflecting the default assignment of the dominant stress pattern.

In contrast, Schiller, Fikkert and Levelt [23] found an advantage for the predominant, as compared to the less frequent stress pattern in picture naming in Dutch. Protopapas et al. [18] and Colombo [11]) found an advantage for the dominant stress in reading aloud, respectively, for Greek and Italian. Thus, as the data do not allow firm conclusions in this respect, one of the aims of the present paper was to find evidence for a tendency to apply the distributionally dominant stress pattern in the language by default.

Further, the possibility to induce stress priming was also investigated in the present paper. This idea has already been explored in the literature, thus far with negative results. Roelofs and Meyer [24] found priming in production task when *both* segmental and metrical information were known in advance. Schiller et al., [23] did not find evidence for stress priming in picture naming with primes of the same or different stress as the targets. In those studies it was assumed that only the less frequent stress pattern – that must be retrieved from the lexicon – can be primed, while the dominant stress, being the default, is not specified, and cannot be primed.

**Figure 1:**
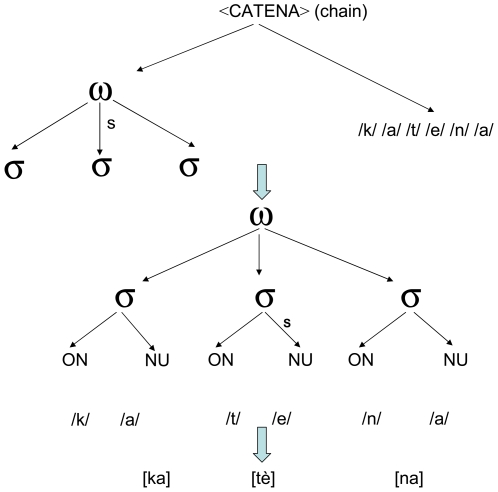
Schematic representation of the metrical representation of stress, and how it connects with the segmental representation during phonological encoding.

In the present study it was assumed that stress is represented as an abstract metrical structure, representing number of syllables and position of stressed syllable. This structure would be associated during the phonological encoding stage to the segmental representation of target words and nonwords activated by the sub-lexical mechanism (see Figure 1, top). If the metrical representation is independent of the segmental one, and if there is a mechanism assigning the stress pattern sub-lexically, it should be possible to prime its application, increasing the likelihood to assign the stress pattern of a target congruently with the prime.

In order to investigate these aspects of stress assignment, the “pathway priming” methodology developed by Zevin and Balota [25] was used, in which a list of five word or nonword primes preceded a target word or nonword. This paradigm was designed to bias the participants to use a lexical or a sub-lexical pathway. In the experiments presented here, target stimuli (words and nonwords) were embedded in lists of either nonwords or low-frequency words with inconsistent stress patterns. Assuming that the extent to which lexical/semantic information is activated can be affected by the context in which the stimulus is presented, and participants can be induced to process stimuli, and to assign stress either sub-lexically or lexically depending on the prime context, the two priming conditions should have opposing influences on performance. In particular, when stress is assigned sub-lexically (nonword primes), stress should more likely reflect the dominant pattern. This would be consistent with the idea of a default mechanism of stress assignment operating as an abstract pattern and reflecting a general bias of the language. Such a bias should not be apparent when stress is assigned lexically, as information about the stress pattern of a specific word is retrieved from the lexicon.

Furthermore, the effect of stress congruence/incongruence of a target with the preceding stimuli in a list was also explored. When stress is assigned lexically, information about the metrical structure of a word is directly available, and there should be no potential for priming effects. In contrast, with nonword primes, favoring a sub-lexical assignment of stress, two contrasting predictions can be made. On one view, the dominant stress might be assigned to the target by default, when its stress pattern is not available from the lexical phonological representation (or is available very slowly, as might happen for low frequency words). In this case a tendency to pronounce targets with the dominant stress should be apparent. Alternatively, the metrical pattern of the prime, specifying position of stressed syllable, might influence the assignment of stress of the target. Thus a priming effect would be expected.

In the experiments of the present study, two prime types were used: a list of words with non-dominant stress, and inconsistent neighborhood, designed to require access to lexical representations, thereby inducing a tendency to assign stress lexically, and a list of nonwords that should have the opposite effect (see Table 2).

**Table 2:** Type of stress pattern used in each condition of the experiments.

Experiment	Word primes	Nonword primes	Word targets	Nonword targets
1	Non-dominant	Dominant	Non-dominant	Dominant
2	Non-dominant	Dominant	Both	________
3	Non-dominant	Non-dominant	Non-dominant	________
4	Both	Both	Both	________

In Experiment 1, the targets were low frequency non-dominant stress words and nonwords. The words required access to the lexical phonological representation to be pronounced correctly, as their neighborhood was formed by dominant stress words, so that neighborhood information was misleading (i.e., they were stress inconsistent nonwords). The nonwords were constructed to include strong cues to dominant stress. Under the conditions of this experiment, the predictions were that if the priming context influenced performance, and access to the lexical representations were less likely with the nonword primes, the bias to assign the dominant stress to target words with non-dominant stress would be stronger in the nonword than in the word prime condition, leading to more stress errors for word targets in the nonword prime condition. As for nonwords, an increase in the proportion of non-dominant stress pronunciations with word- as compared to nonword- primes would be evidence of stress priming produced by the prime words.

Experiment 2 was a replication of Experiment 1, in which the nonword targets were replaced with low frequency dominant stress words (see Table 2). The aim of the substitution was to see if the increase in stress errors for words in the context of nonword primes obtained in Experiment 1 depended on the stimuli being words, rather than nonwords, or depended on the stimuli having non-dominant stress. It could be that applying stress sub-lexically generically increases the probability of errors for words. On the other hand, if the pattern observed in Experiment 1 were the result of a tendency to apply a default, or of stress priming, that would predict no increase in errors in the nonword context for dominant stress word targets with a stress pattern homogeneous to the primes.

A further prediction, tested in Experiment 3, would be that if non-dominant stress targets were preceded by nonwords homogeneous for stress, no decrement in performance should be found. Thus, in Experiment 3 high and low frequency non-dominant stress words were presented primed by words and nonwords with the same stress (see Table 2).

In Experiment 4, the stress patterns of the context stimuli were heterogeneous, thereby eliminating the possibility that primes created a dominant pattern at a local level producing stress priming. The targets were the same as in Experiment 2, that is, dominant stress - consistent words, and non-dominant stress -inconsistent words, and word primes were dominant and non-dominant stress items in an equal proportion (see Table 2). The word prime context should provide a strong incentive to attend to lexical information, because the items themselves require whole-word processing, and the context does not provide any strong local cues to stress as in Experiments 1 and 2. The nonword primes were drawn from highly consistent neighborhoods. Half of them had a high probability of being assigned dominant stress, and half non-dominant stress. For target words with less frequent stress an increase in the proportion of stress errors should be found, replicating the previous results. For dominant stress words, if the activation of sublexical codes is more likely with nonword primes, and stress on the penultimate syllable applies as a default, the same pattern as in Experiment 2 should be found, with no decline in performance in the nonword priming condition. If an increase in errors were found, however, this would weaken the idea of a default assignment of stress.

## Methods

### Ethics Statement

All the experiments of the present study were conducted in accordance with the policies of the Ethics Committee of the University of Padua, Faculty of Psychology; participants provided oral informed consent, as the test was completely anonymous.

### Experiment 1

#### Participants

Twenty-four students of the University of Padua participated in the experiment.

#### Materials

Eighty-four three-syllabic words with stress on the antepenultimate syllable were selected from a corpus of 1,500,000 occurrences (mean frequency = 2.54; range = 1–10; Corpus di Barcelona, Istituto di Linguistica Computazionale, 1989, unpublished manuscript). These are non-dominant stress words, which are *inconsistent* because their neighborhood is formed by a large majority (73.71%) of words with the same final syllables (more precisely, the nucleus of the penultimate syllable and the last syllable) and a pronunciation with the *dominant* stress pattern (i.e., stress assignment on the penultimate syllable; see [11], [12] for details). The stress assignment of this neighborhood is thus inconsistent with that assigned to the target stimuli, leading to longer naming latencies and to regularization errors in unprimed naming. Tables 1 and 2 display examples of words in the different conditions of stress and consistency. Only the dominant consistent and the non-dominant inconsistent words were used in the present study.

The set of 84 words was used to create the lists of primes and targets (see Appendix S1). To the set of words a set of 84 nonwords was added. These were three-syllabic letter strings, designed to have strong neighborhood cues to the dominant stress pattern. As shown in Colombo [11], [12], word neighborhood is a good predictor of the type of stress assigned to nonwords. Thus, in order to ensure that the dominant stress pattern was assigned to nonwords, they were constructed to include as an ending the nucleus of the penultimate syllable and the last syllable of words stressed with the default pattern (on a pre-test, on the average 81% of these nonwords had a dominant stress pattern).

Two types of list were made. One list type contained 70 non-dominant stress words to be used as primes, and 14 non-dominant stress words to be used as targets. The second list type included 70 nonwords primes and 14 nonword targets. Each list type of 84 items was formed by 14 mini-blocks, each formed by 5 primes and 1 target. The stimuli were divided between the two lists types so that in each list there were 7 word targets and 7 nonword targets. Primes and targets were matched for initial phonemes between lists.

Six blocks were constructed based on two lists, one with non-dominant stress word primes and one with nonword primes. Each stimulus occurred as a prime in each position, and as a target. Stimulus order was counterbalanced across blocks. Each participant was assigned a block composed of the two lists, one with 70 words with non-dominant stress as primes and 7 words and 7 nonwords targets, the second with 70 nonwords primes and a different set, but the same number, of words and nonwords as targets. The order of each list was counterbalanced across participants. The sequence of stimuli consisted of five primes and a target, in an uninterrupted list, so that participants were not aware of the status of the stimuli as primes or targets. Half of the participants saw the word primes list first, while the other half was first presented the nonword primes list. A list of 12 different words and nonwords were used as practice trials.

### Experiment 2

#### Participants

Thirty-six students of the University of Padua served as participants in this experiment. None of them had been a participant in Experiment 1.

#### Materials

Design and structure of the experiment were the same as in Experiment 1. Eighty-four three-syllable low frequency words with dominant stress were selected, with a frequency range 1–26 (mean = 7.43) and 88% consistent neighbors (i.e., neighbors with the same ending and the same stress pattern). These words replaced the nonword targets in the lists used in Experiment 1, while the non-dominant words were the same. Two list types were created. One list contained 70 words to be used as primes, and 14 words to be used as targets. The second list included 70 nonword primes and 14 word targets. In each list there were 14 mini-blocks composed of 5 primes and 1 target in succession. In each list there were 7 low frequency dominant stress word targets, and 7 low frequency non-dominant stress word targets. Primes and targets were matched for initial phoneme. There was an attempt to match initial consonants between sets of dominant and non-dominant stress stimuli.

Block composition was the same as in Experiment 1, except that the set of dominant stress words was only used for target words, by assigning seven dominant stress words to each of the six two-lists blocks. Each participant was assigned one block constructed from the two lists, one with words with non-dominant stress as primes and 7 low frequency non-dominant stress words and 7 low frequency dominant stress word targets, the second with nonword primes and a different set, but the same number, of non-dominant and dominant words as targets. The order of each list was counterbalanced among participants so that half of the participants saw the non-dominant stress word prime list first, while the second half of the participants was assigned the nonword primes list first. A list of 12 non-dominant and dominant stress words, not present in the experimental lists, was used as practice trials.

### Experiment 3

#### Participants

Thirty-six volunteer students of the University of Padua participated in Experiment 3.

#### Materials

Eighty-four three-syllabic words were selected, 42 low frequency and 42 high frequency non-dominant stress words (mean = 4.6, range 1–17 for low frequency words; mean = 109.7, range = 32–395, for high frequency words; Corpus di Barcelona, 1989). These words have a majority (low frequency = 77.50%, high frequency = 68.4%) of stress inconsistent neighbors, (words with the same final spelling pattern *but* a dominant stress pattern).

The prime words were the same as used in Experiments 1 and 2. A new set of 70 nonwords was selected from an existing database (derived from previous unpublished experiments of the first author), including nonwords that were pronounced by the large majority of participants with stress on the initial syllable (i.e., with a “non-dominant” stress). This was done by creating nonwords in which the nucleus of the penultimate syllable and the last syllable were taken from words with the same spelling pattern and a non-dominant stress pattern (the average percentage of non-dominant stress pattern for these words being 74%, based on a type count). Former studies have demonstrated that this manipulation is efficient in inducing a non-dominant stress pattern in nonword pronunciation [11], [12]. Indeed, neighborhood consistency determined 77% of non-dominant stress assignment for this set of 70 nonwords in a pre-test.

The lists composition was the same as in the previous experiments. The stimuli were divided between the two lists so that in each list there were 7 low frequency and 7 high frequency non-dominant stress word targets. Each target was presented in both conditions, with word and nonword primes, but in different blocks, so as to avoid effects caused by the repetition of the same stimulus in the two lists.

Each participant was assigned a block composed of the two lists, one with words with non-dominant stress as primes and 7 low frequency and 7 high frequency non-dominant stress word targets, the second with non-words primes and a different set, but the same number, of low and high frequency words as targets. The order of each list was counterbalanced among participants in the same way as in the previous experiments. Finally, a list of 12 different low and high frequency words with non-dominant stress was used as practice trials. The procedure and equipment were the same as in the preceding experiments.

### Experiment 4

#### Participants

Forty-four students of the University of Padua participated in the experiment.

#### Materials

The targets used in Experiment 4 were the same dominant consistent and non-dominant inconsistent stress words of Experiment 2. One-hundred sixty-eight words were selected to be used as primes, 84 with dominant stress, and 84 with non-dominant stress. The non-dominant stress primes were the same as used in the former experiments, while the dominant stress words were selected from the set of low frequency words of the Corpus di Barcelona frequency norms.

The nonword primes were formed by 168 pronounceable nonwords. Half of these nonwords (84) were the same as used in Experiments 1 and 2 as primes and had a high probability of being named with dominant stress. The other half of nonwords (84) was selected from an existing database (from unpublished experiments of the first author) and had a high probability to be named with non-dominant stress. That is, the non-dominant stress nonwords were constructed with an ending that was present in word neighbors with non-dominant stress, (the average percentage of the non-dominant stress pattern for these words being 74%). The nonwords were selected after a pre-test in which reading aloud latencies were collected in a group of participants that was not tested in any of the other experiments. Lupker, Brown & Colombo [26] showed that there may be list composition effects on reading latencies, as a consequence of a tendency of subjects to homogenize latencies, such that when fast stimuli are presented in pure lists, they are faster than when mixed with slow stimuli. The latency results of Experiments 1 and 2 are not consistent with an interpretation in terms of this homogenization tendency (time criterion). However, in Experiment 3 we decided to avoid the possibility of confounding factors, therefore we equated the latencies for the two types of primes, selecting nonword primes as fast as low frequency words, on average.

Six blocks were formed. Each block was made up of two lists. Each word prime list had an equal number of dominant and non-dominant stress words as primes (35). The list with nonword primes was composed of 35 nonwords with a high probability to be named with dominant stress, and 35 with non-dominant stress, based on the stress neighborhood information. In each list there were 7 low frequency dominant consistent and 7 low frequency non-dominant inconsistent word targets. Each participant was assigned one block constructed from the two lists, one list formed by 70 prime words and 14 targets words, one composed of 70 nonwords and 14 word targets. The order of each list was counterbalanced across participants as in the previous experiments. Finally, a list of 12 different dominant and non-dominant words and nonwords was used as practice trials. The procedure and equipment were the same as in the preceding experiments.

#### Procedure

A PC Pentium 75 Mhz processor running in DOS mode controlled the experiment. The monitor was in color VGA. A voice key connected to the PC's real-time clock was used to collect response latencies and response durations to the nearest ms.

Stimuli were presented on the screen of a computer monitor. A white asterisk was presented for 400 ms, followed by the stimulus, that was colored blue after 300 ms from the onset, and remained on the screen for 1800 ms. After such period, or at the start of articulation, the letter string disappeared and was followed by the naming latency for that trial. If the participant wasn't able to respond within this time limit, the trial was removed from the analyses. The experimenter coded each trial as correct or as an error, and in the latter case the type of error was recorded. The inter-trial interval was 1400 ms. Participants were instructed to read the words aloud, trying to be as fast and accurate as possible.

## Results

### Experiment 1

In this and the following experiments, the predictions and the discussion of the results will be centered on the pattern of errors, rather than on RTs, although the results of the latter are reported for completeness. Recent studies have shown that voice keys introduce measurement problems in detecting the onset of multisyllabic stimuli (e.g., [27]). In particular, voice onset latencies might differ for tonic syllables as compared to unstressed syllables, and this fact requires some caution in interpreting RT results. Although the main interest of the study lies in comparing the same stimuli in different contexts, which means that the acoustic measurement problem is the same in both conditions, we prefer to be cautious and discuss mainly the error pattern. Moreover, the main interest of the present study was in the type of stress participants would assign depending on whether reading was lexically or sub-lexically driven, and in the conditions under which stress errors were committed.

The data were mean correct naming latencies and mispronunciation errors, displayed in Table 3. Latencies below 200 ms and above 1800 ms (about 1.1%) were automatically removed. Stress errors, i.e., pronunciations with the dominant pattern of words with non-dominant stress, and vice versa (i.e., pronunciations with the non-dominant stress of words with the dominant stress) were analyzed separately from mispronunciation errors. Mean latencies and error percentages are displayed in Table 3.

**Table 3:** Mean correct naming times, percentage of stress errors (in parentheses) for word targets with non-dominant stress, primed by words with non-dominant stress and nonwords with dominant stress in Experiment 1 (above).

Prime Type	Nonword prime - N-Dom	Nonword prime-Dominant
Targets	RT	RT
N-Dom Stress Words	582 (3.57%)	608 (13.7%)
Nonwords	649 (69%)	618 (76%)

Mean latencies and percentage dominant stress assignment (in parentheses) to nonword targets, under the two priming conditions (below).

N-Dom. Stress words = Non-dominant stress words.

Word prime- N-Dom = Word prime Non-Dominant stress.

#### Errors

The analysis of stress errors to word targets (i.e., non-dominant stress words pronounced with the dominant stress, i.e. “regularisation” errors) showed that, in agreement with the predictions, these errors were significantly more likely after nonword primes (13.4%) than after word primes (3.6%), [t_1_ (23) = 5.02, p<.001; t_2_ (82) = 2.44, p<.05]. The analysis of mispronunciation errors (0.03%) was not carried out because of too many empty cells.

Nonword targets were predominantly assigned dominant stress, with a higher percentage of dominant stress assignments after nonword primes (76%) than after word primes (69%), although the effect of prime type was not significant. Finally, the percentage of dominant stress assignment was 76% on nonword primes and 2.74% on non-dominant word primes. Mispronunciation errors were few (0.42%).

#### Latencies

The analysis of variance was conducted with both participants (F1) and items (F2) as random variables. The design included two factors, prime type and target type. The ANOVA on RTs showed a marginally significant main effect of target type, only by subjects [F_1_(1,23) = 3.89, MSe = 2546.87, p<. 1; F_2_ ns], while the main effect of prime type was not significant. The interaction was significant [F_1_(1,23) = 5.37, MSe = 1495.73, p<.05], F_2_(1,164) = 3.87, MSe = 7011.75, p = .05), with target words faster after word- than nonword primes (t_1_(23) = 1.93, p<.07; t_2_ (82) = 1.48, ns) while nonwords showed the opposite trend. In the analysis of prime latencies there was a significant effect of prime type (38 ms), [t_1_(23) = −3.46, p<.01, t_2_(166) = 4.61, p<.001], with word primes (584 ms) faster than nonwords (622 ms).

### Experiment 2

As in Experiment 1, the data were mean correct naming latencies, mispronunciation and stress errors. Latencies below 200 ms and above 1800 ms (about 1%) were automatically removed from the RT analyses. The latency data for one item were lost due to experimenter error.

#### Errors

There was a prime type effect: stress errors were significantly more frequent after nonword primes than after word primes, F_1_(1,35) = 10.92, MSe = .008 p<.01, and F_2_(1,164) = 6.09, MSe = .014, p<.05. There was also a main effect of type of stress, F_1_(1,35) = 15.33, MSe = .006, p<.001, F_2_(1,164) = 6.62, MSe = .014, p = .05. The interaction was significant, however, F_1_(1,35) = 8.79, MSe = .007, p<.01, F_2_(1,164) = 4.56, MSe = .014, p = .05, showing an effect of prime type on non-dominant stress words (see Table 4). A t test comparison confirmed that the effect of prime type was only reliable in the non-dominant stress condition, (t_1_(35) = 3.66, p<.01; t_2_(82) = 2.59, p<.05). Mispronunciation errors were few (0.02%) and the analysis was not carried out because of empty cells.

**Table 4:** Mean correct naming times and stress error percentage (in parentheses) for low frequency word targets with non-dominant and dominant stress, primed by words with non-dominant stress (ND) and nonwords in Experiment 2.

Prime Type	Word prime-Dominant	Nonword prime-Dominant
Targets	RT	RT
N-Dom Stress Words	601 (3.17%)	588 (12.30%)
Domin Stress Words	606 (2.38%)	573 (3.17%)

N-Dom. Stress words = Non-dominant stress words.

Domin Stress Words = Dominant stress words.

Consistently with the data of Experiment 1, more dominant (86%) than non-dominant stress (14%) pronunciations were assigned to nonword primes. Mispronunciation errors were 0.2% on nonword primes and 0.5% on word primes. “Regularization” stress errors on word primes were 3%.

#### Latencies

In the analysis of latencies, the effect of prime type was only marginally significant by participants [F(1,35) = 3.88; MSe = 4718.14, p = .057; F(1,163) = 2.45, ns]. Low frequency dominant stress word targets were faster (30 ms) when primed by non words, compared to when primed by low frequency non-dominant stress words, [t_1_(35) = 2.72, p<.05; t_2_ (82) = 1.92, p = . 06]. The effect on low frequency non-dominant words was not significant (t<1). Neither the main effect of stress, nor the interaction were significant. In the analysis of prime latencies, the 9 ms difference between word (604 ms) and nonword (613 ms) primes was not significant [t_1_ (35) = 1.03; t_2_ (166) = 1.45].

### Experiment 3

#### Errors

No analysis of stress “regularization” or mispronunciation errors (1.4%) was carried out on targets because there were too many empty cells. The analysis on nonword primes confirmed that the majority of nonword primes were pronounced with non-dominant stress (96%), showing the successful manipulation of stress due to neighborhood. Mispronunciation errors were 0.95% on word primes, 3.5% on nonword primes. A small percentage (3.3%) of non-dominant word primes were pronounced with dominant stress.

#### Latencies

Latencies below 200 ms and above 1800 ms (0.9%) were automatically removed from analyses. The pattern of both latencies and errors showed clearly no effect of prime type (see Table 5). In the ANOVA on target latencies, the only significant effect was that of frequency, [F_1_ (1,35) = 9.610, MSe = 1843.78, p = .004; F_2_ (1,82) = 4.54, MSe = 4499.95, p = .036]. In the analyses of prime latencies, there was a small (14 ms) but significant difference [t_1_ (35) = −1.9, p = .066; t_2_ (138) = 3.806, p<.000], with word primes (626 ms) slower than nonwords (612 ms).

**Table 5:** Mean response latencies (RT) as a function of prime type and target type and percentages of stress errors (in parentheses) in Experiment 3.

Prime Type	Word prime- N-Dom	Nonword prime- N-Dom
Targets	RT	RT
Low F N-Dom. Stress	591 (2.38%)	584 (1.98%)
High F N-Dom. Stress	564 (0.40%)	567 (0.40%)

Low F N-Dom Stress = Low frequency words –Non dominant stress.

High F N-Dom. Stress = High frequency words –Non-Dominant stress.

### Experiment 4

#### Errors

The pattern of errors revealed a strong effect of prime lexicality on performance. The overall proportion of errors was greater in the nonword prime condition (0.17) than in the word prime condition (0.09), a significant effect, F_1_(1,43) = 15.73, MSe = 0.017, p<.001; F_2_(1,164) = 7.87, MSE = 0.032, p<.01. There was also an interaction, such that this effect was greater for non-dominant stress items than for dominant stress items, F_1_(1,43) = 9.47, MSe = 0.017, p<.01; F_2_(1,164) = 4.76, MSe = 0.032, p<.05. A-priori comparisons revealed that the effect of prime type was significant in the non-dominant stress condition, t_1_(43) = 5.86, p<.001, t_2_(82) = 3.56, p = .01, but not in the dominant stress condition, t<1.

Considering in particular stress errors, the proportion of such errors was greater in the nonword prime condition (0.15) than in the word prime condition (0.06). This effect was significant, F_1_ (1,43) = 26.35, MSE = 0.013, p<.001; F_2_((1,164) = 12.38, MSE = .028, p<.01. The interaction between priming condition and stress pattern of targets was not significant (by subjects, F(1,43) = 3.66, MSE = 0.01, <.1; by items, ns). Separate t-tests showed that the prime type effect was significant in the non-dominant condition (t_1_(43) = 5.29, p<.001; t_2_(82) = 3.23, p<.001) and significant by participants in the dominant stress condition (t_1_(43) = 2.59, p<.01; t_1_(82) = 1.74, .05<.1).

Finally, an analysis of errors on primes showed an effect of lexicality: the proportion of errors was 0.12 on word primes, 0.27on nonword primes, t_1_(43) = 10.62, p<.001; t_2_(334) = 8.09, p<.001. The proportion of stress errors was 0.07 on word primes. Overall the proportion of dominant stress pronunciations on nonwords was 0.42; 0.70 of the “dominant stress” nonwords received a dominant stress, while only 0.19 of the “non-dominant stress” nonwords received a dominant stress, showing that the manipulation of stress neighborhood on nonword stress pattern was fairly successful.

#### Latencies

The analysis of variance on target latency was first carried out on correct latencies, after removing invalid trials (1%) and outliers (i.e., latencies below 200 ms and above 1800 ms, 1.6%). As shown in Table 6, there were no significant effects of prime type, of target type, or any interaction (all F's<1) in the analysis of latencies. Correct response latencies for primes were 644 ms for words and 646 ms for nonwords, t_1_(43)<1; t_2_(334)<1.

**Table 6:** Mean response latencies (RT) as a function of prime type and target type and percentages of stress errors (in parentheses) in Experiment 4.

Prime Type	Word prime	Nonword prime
Targets	**RT**	**RT**
N-Dom. Stress Words	619 (4.92%)	624 (16.70%)
Domin. Stress Words	615 (6.87%)	626 (12.90%)

N-Dom stress words = Non-dominant stress words.

Domin Stress Words = Dominant stress words.

## Discussion

### Experiment 1

The pattern of stress errors obtained in Experiment 1 showed that when primes were nonwords, there was a stronger tendency to assign dominant stress to words with non-dominant stress, committing a stress error. In contrast, when the primes were words requiring access to the lexical output representations, stress errors were very few. Nonwords were assigned dominant stress, and were not significantly affected by the prime type. This result suggests that the manipulation successfully induced a tendency to assign stress lexically or sub-lexically depending on prime lexicality.

The effects obtained in Experiment 1 are consistent with two possible explanations, as regards the error increase with nonword primes in Experiment 1. The first is that when stress was assigned sub-lexically, and the lexical phonological representation was not available quickly, the default dominant pattern applied, as mentioned earlier. The second is that there was stress priming, and because nonwords were mainly pronounced with the dominant pattern, this led to pre-activation of that pattern.

### Experiment 2

In both Experiment 1 and 2 the proportion of stress errors for inconsistent non-dominant stress words increased in the context of nonwords relative to a word context. In contrast, there was no increase in stress errors for dominant stress targets preceded by nonword primes. These results can be interpreted as evidence that decreased lexical activation with nonword primes increased the probability of assigning the dominant stress, and of making stress errors on words with the less frequent stress pattern. This effect might be exaggerated by the fact that nonword primes were mainly stressed on the penultimate syllable, which is the most frequent stress pattern in the language, therefore producing a bias in favor of dominant stress. According to this interpretation, when the sub-lexical mechanism prevails, the stress pattern will be assigned by imposing the default, and there should be little or no effect of whether the stress pattern of nonword primes is congruent or incongruent. So there should be an increase in latency and errors for non-dominant stress words, independently of the stress pattern of nonword primes.

According to an alternative interpretation, word targets were simply facilitated by the prior presentation of a prime (word or nonword) with *the same* stress pattern. Thus the source of penalization was the presence of a context with a prosodic pattern that was not homogeneous with the target word itself. This interpretation lead to the prediction, tested in Experiment 3, that if non-dominant stress targets were preceded by nonwords homogeneous for stress, no decrement in performance should be found.

### Experiment 3

The results of Experiment 3 showed that there was no increase in the proportion of errors for non-dominant stress words when preceded by nonwords pronounced with the same stress pattern. Thus, words with the less frequent stress were read more slowly and less accurately *only* when preceded by nonword primes with dominant stress (Experiments 1 and 2), whereas they were not penalized, when preceded by nonword primes that shared their stress pattern. These findings support an interpretation of the data of Experiments 1–3 in terms of stress priming, (i.e., a tendency to homogenize the stress pattern assigned to a word to that of a list context). As the increase in errors was only found in the nonword prime context, this finding suggests that stress priming can be obtained only when stress is assigned through a sub-lexical mechanism.

These results, however, raised the question whether a default mechanism biasing the assignment of the most frequent stress pattern (on the penultimate syllable) indeed exists. Clearly such mechanism is used in assigning stress to nonwords. However, little evidence was found for an effect of this mechanism on words. One possibility to explain this result is that the five homogeneous stress primes formed a strong local context that counteracted the tendency to apply a default. One way to verify this interpretation is to attenuate the local bias given by homogeneous stress items, by presenting prime stimuli with mixed stress pattern types. This was done in Experiment 4.

### Experiment 4

The results of Experiment 4 were clear. In the nonword prime condition, by mixing dominant and non-dominant stress items, the proportion of stress errors considerably increased, partially replicating the effects of Experiments 1 and 2. In particular, the manipulation of mixing the stress patterns of both word and nonword primes yielded both “regularizations” and “irregularizations”. The presence of irregularization errors, despite the consistency of neighbors of dominant stress words, is noticeable, as these were the same words that in Experiment 2 did not elicit but very few errors. Thus, the error increase can only be attributed to the effect of context, where priming nonwords had the same probability, according to stress neighborhood, to be assigned dominant or non-dominant stress. This is also reflected in the fact that the average proportion of dominant stress pronunciations assigned to prime nonwords was only 0.40, showing that participants did not tend to assign dominant stress by default (see, in comparison, the proportion of 0.86 dominant stress obtained in Experiment 2, where nonwords were selected to be named with dominant stress). These results, in particular the increase in stress error rate, can be explained assuming that participants were less likely to consult lexical information with nonword primes, and so they were more likely to make stress errors, even for dominant stress items. The results are consistent with data by Burani and Arduino [14], suggesting little evidence for an advantage of the dominant stress pattern. They found stress neighborhood consistency effects on both dominant and non-dominant stress words. They even found a reversal of the stress effect, with faster latencies for non-dominant stress words with a higher number of consistent neighbors, as compared to the dominant stress words. If dominant stress were applied as a default, one would have expected slower latencies for non-dominant stress words.

The effect of neighborhood consistency on nonwords was very robust in the present study. We were able to create nonwords pronounced predominantly with the less frequent stress pattern on the basis of stress neighbors, confirming the findings by Colombo [11]. Moreover, the stress neighborhood manipulation on nonwords was so strong as to produce a large number, not only of “regularization” errors, but also of “irregularization” errors.

### Overview of Experiments 1–4

In the Introduction, it was suggested that three independent sources provide information to assign lexical stress in reading Italian aloud: Lexical (speakers know the stress patterns of particular words), metrical (speakers know what stress pattern is most common in their language) and an intermediate “sub-lexical” level (speakers are sensitive to the statistical relationships among particular segmental patterns and stress). In the four experiments reported here, the contribution of each of these sources of information was modulated by priming context in a word naming task.

Evidence that application of a metrical frame can be modulated by context comes from Experiments 1-3. Priming non-dominant stress words with dominant stress nonwords resulted in a large proportion of regularization errors, relative to priming with non-dominant stress words. On our view, these results suggest that the repeated application of a particular stress pattern can prime the stress pattern itself.

Evidence that the role of lexical and sublexical information in reading aloud can be modulated by priming comes from Experiment 4. In this experiment, no specific stress pattern was cued, as both priming lists contained words with both stress patterns. In the word priming condition, lexical activation was emphasized, whereas sub-lexical cues to stress were emphasized in a priming condition with consistent nonwords. Because both priming conditions contained items of both dominant and subordinate stress, there was no dominant pattern at the list level, in contrast to the earlier experiments, in which each list included prime stimuli with a homogeneous stress pattern (i.e., dominant stress for nonwords; subordinate stress for words). Nonetheless, large differences in performance were observed between the two priming conditions. In particular, a much larger proportion of stress errors was observed in the nonword prime condition, suggesting a de-emphasis of lexical information. This was somewhat modulated by the dominant metrical frame and by the consistency of sublexical cues: “regularization” errors, in which the dominant stress pattern was incorrectly applied, were more frequent than “irregularization” errors (in which the subordinate stress pattern was incorrectly applied).

Thus, overall, the experiments of the present study show evidence for three important aspects related to the processing of prosodic information in reading. First, stress can be primed under certain conditions (Experiments 1–3). Second, stress can be assigned sub-lexically. Third, there is weak evidence for a default assignment of the dominant stress to words, and only when the sublexical mechanism is used (Experiments 2 and 4).

Although we have described the phenomena observed here only in terms of dual-process models for convenience, they are also consistent with models in the “triangle” framework [3]–[5]. In such models, mappings from orthography to phonology are encoded at multiple levels of description, ranging from single letters or graphemes to whole words. Additional information about whole words is available during spelling-to-sound translation from mappings via semantics. Thus, for words with highly unusual spelling-to-sound correspondences, task parameters that bias performance toward direct mapping from spelling to sound can result in regularization errors [25]; see [28] for an alternative interpretation of this finding).

There is no model of Italian reading in the triangle framework, although extensions to other languages (e.g., Chinese, [29], [30]) suggest that the notion of extracting regularities at multiple grain sizes is not idiosyncratic to grapheme-to-phoneme translation, and applications of similar models to the computation of stress from spelling (Zevin & Joanisse, unpublished manuscript; [31]) suggest that probabilistic cues to stress are available cross-linguistically and can be learned by the same mechanisms that underlie spelling-to-sound correspondences at the segmental level. In such a model, the dominant stress pattern in the language would be encoded as a bias to produce that stress pattern, based on its frequency. This bias could be over-ridden by sublexical cues to stress (i.e., neighborhood statistics), or on the basis of whole-word information encoded both in direct and semantically-mediated pathways.

This model would explain the stress priming results in more or less the same way as they are explained in a dual-process framework: The stress pattern is part of an output representation, and its resting level of activation can be modified by naming items with the same stress pattern. Stress errors observed under conditions of nonword priming could be accounted for by increased gain on the direct conversion of spelling-to-sound, which would be adaptive for naming nonwords (i.e., more efficient performance for the direct, as compared to semantically mediated, orthography –phonology conversion, and for smaller, as opposed to larger size units). Just as words with unusual spellings depend probabilistically on semantic input in models of monosyllabic word reading, we should predict stress errors under nonword naming as a result of the interference of sublexical cues to stress that compete with whole-word information in the direct mapping from spelling to sound.

### Stress priming

An important finding of the present study was that when sub-lexical codes prevailed, and there was a homogeneous stress context (Experiments 1–3), the stress pattern of a word could be primed, suggesting the existence of a mechanism that operates as an abstract representation on a syllabically segmented tier in which the phonemes are not specified. The evidence of prosodic priming supports the idea that stress can be computed independently of segmental information [7], [11].

The current results contrast with Schiller et al.'s [23] study, in which a stress priming effect was not found. The first aspect to consider is that the task used in Schiller et al. was picture naming, while in the present work, subjects read aloud. In a picture naming task, the process of phonological, and subsequently metrical encoding, may be based on lexical phonological representations. In addition, their primes were all words, further reducing the possible influence of sub-lexical information, while in the present experiments an increase in stress errors was found with nonword primes.

Overall, the data suggest that stress priming does occur, but its origin is not lexical, that is, it is not produced by priming of words with the less frequent stress by phonological representations of consistent words. Instead, the present results are congruent with the idea that there is a separate representation of stress, which has been implicitly learned by speakers of a language, and abstracted from the segmental context. Such structure can be primed under certain conditions, in the same way as the rhythmic structure of music can [32]. This view is congruent with the idea that there are similar organizing principles in the linguistic and musical domains [33].

### Implications for models of word reading and production

The present results are obviously relevant for modeling the interface between word naming and speech production, with the inclusion of stress assignment, which is rather neglected in existing computational models. Up to now, the most complete attempt to include stress in a model of reading was made by Rastle and Coltheart [10] on English disyllabic words, with a modification of the DRC model, to include a number of rules that identify prefixes and suffixes, and assign stress consequently. This procedure raised the question whether a stress pattern can be assigned by completely non-lexical rules in English. This issue is extremely relevant in the present context, given the proposed interpretation of effects obtained with nonword primes. In particular, one characteristic of stress neighborhood, that up to now has not been investigated, is the fact that many stress neighborhood endings are formed by morphological elements. For example, the endings “-ale” and “-ino” in the nonwords “erale” and “ ammino” are derivational suffixes that attract stress, whereas “-ano” in “gofano” is an inflectional suffix not attracting stress. A strong implication might be that stress neighborhood effects are morphologically determined. Further implication for the present study might be that effects induced by nonword primes including morphological affixes might not be derived as output from a sublexical mechanism. In contrast, they might be derived by activation of lexical units containing endings corresponding to morphemes. In agreement with Rastle and Coltheart [10] this would blur the distinction between lexical and sublexical routes, and require the two mechanisms to interact. One possible locus of interaction would be the phonological buffer, in the more recent version of the DRC (Dual Rote Cascaded) model, where outputs of the two routes are stored [2]. This model is able to simulate pattern of data in nonword reading, for example pseudohomophone effects that are explained by the interaction of the two routes, while still assuming that nonwords are named via the sublexical mechanism.

An alternative explanation, in terms of a triangle model, would be that there is no special representational status of morphemes, and that they are emergent properties of learning relations among phonology, orthography and meaning [34]. Word endings that have been frequently associated with an unstressed realization will tend to activate phonological (and phonetic) units consistent with them, and the opposite is the case for units associated with phonologically stressed realizations. In this way, there would be no differentiation among morphological attractors of stress (i.e., derivational suffixes), and units not attracting stress (i.e., clitics), and non-morphological endings, and stress neighborhood effects would be conceived as emerging from statistical correlations between orthography and phonology. Moreover, as suggested above, stress neighborhood with nonword primes would be the result of an adaptive performance incrementing orthography-phonology weights to small -sized units.

Given the regularity of spelling to sound correspondence in Italian, it is conceivable that stress assignment can be computed sub-lexically in this language. How this operation is interfaced with the computation of phonology from orthography remains to be specified. In agreement with Miceli and Caramazza [22], it can be assumed that the output of the orthography–to-phonology conversion mechanism is a phonological representation that is syllabically specified. Moreover, the syllable can be endowed with a specification of whether it includes a tonic vowel or not, when sufficient information is available either from the syllable structure (a closed penultimate syllable), or from neighborhood. In both cases, the orthographic cluster forming the nucleus of the penultimate syllable and the last syllable of a consistent neighborhood would activate a phonetic representation in which the characteristics of the vowel of the penultimate syllable (i.e., whether it is a tonic vowel or not) are already specified. For example, the non-lexical procedure for words or nonwords including an ending like –ATO (whose neighborhood is formed by dominant stress words) would output a sequence of segments in which the stress-carrying vowel of the unit –ATO would be characterized by longer duration and intensity. On the other hand, if the ending of a word is –OLO (a unit found in words with non dominant stress), the sequence of segments that would be activated would not include a tonic vowel, therefore the first vowel of –OLO would be characterized by short duration and low intensity. This specification in turn might be used as a cue to shift the position of stress on the preceding syllables (i.e., if the penultimate syllable does not carry stress, stress position should be shifted to the initial syllable).

How the specification of stress is computed when this type of information is unreliable or insufficient (for example, when there is no dominant neighborhood, or no information from the syllable structure) and the output of the spelling-sound conversion must be interfaced with lexical information requires further explorations. The proposal that under such conditions the default stress assignment is applied does not seem so straightforward, given the limited effect of dominant stress found in the present study, and the fact that normally speakers and readers do not make many stress errors in producing words (in particular, those favoring dominant stress). However, the evidence for stress priming when sub-lexical mechanisms dominate confirms the idea that stress can be represented separately from lexical and segmental information, and that this representation is sensitive to the effect of context at a very general, or at a local level.

## Supporting Information

Appendix S1 Lists of stimuli used in Experiments 1–4.
(0.04 MB DOC)
