# Supplementary material for: Stress Priming in Reading and the Selective Modulation of Lexical and Sub-Lexical Pathways
Source: PLoS One. 2009 Sep 29;4(9):e7219. doi: 10.1371/journal.pone.0007219 (PMC2747276; doi:10.1371/journal.pone.0007219)
Supplement: Appendix S1 — Lists of stimuli used in Experiments 1–4. (0.04 MB DOC) [file pone.0007219.s001.doc]

**Appendix S1**

Low frequency words with non-dominant stress (on the antepenultimate syllable) used in Experiment 1:

Afona, Agave, Alluce, Andito, Angora, Argani, Argine, Augure, Bibita, Bifore, Bigamo, Bipede, Bisturi, Bosforo, Bretone, Canfora, Carpino, Celtica, Cerberi, Cernita, Concavo, Crimine, Darsena, Decadi, Delega, Despota, Diafane, Dollaro, Edito, Egadi, Egloga, Esodo, Femore, Fiocine, Folaga, Folgore, Forfora, Fosforo, Fradice, Gamberi, Inclito, Indaco, Inferi, Inguine, Lamina, Lecito, Ligure, Logoro, Macina, Malaga, Mantice, Marmore, Onice, Orfani, Panama, Patina, Pomice, Postuma, Prodigo, Profuga, Proroga, Recluta, Redini, Remore, Rendita, Ricino, Ruderi, Satiro, Satura, Scomodo, Stipite, Suddito, Sudice, Talamo, Talleri, Timpani, Tonaca, Tortora, Totani, Vortice, Zattera, Zefiro, Zigomo, Zingaro.

Nonwords used in Experiment 1:

Adoso, Aldume, Alona, Ammino, Arfina, Astone, Berino, Bildese, Birtona, Bistone, Bodune, Botaro, Bovato, Calave, Caleri, Calune, Cecana, Compela, Crimito, Delore, Denora, Deturo, Dilaro, Dinuro, Disena, Efede, Ellate, Epede, Erale, Etaso, Fedale, Fegore, Fevone, Forlata, Frellata, Frotona, Gamito, Gatera, Incroni, Iruta, Issubo, Iviero, Lantiro, Lenolo, Lidame, Linata, Mafona, Mascato, Mavino, Mevino, Mimore, Olina, Onese, Ostore, Pidoso, Parede, Paroga, Pifato, Polaso, Povato, Raddame, Regito, Retiro, Rifuta, Rivino, Rudomi, Sentana, Sintura, Solume, Sorato, Stofore, Subela, Tegoro, Tentoro, Tevone, Tirloni, Tomuro, Tuposo, Vabore, Vernite, Zaloci, Zilaso, Zilota, Zolita.

Words with dominant stress (on the penultimate syllable) used as targets in Experiment 2:

Aceto, Afoso, Albume, Androne, Arnese, Arsura, Bagnino, Bendato, Berlina, Bitume, Blasone, Borgata, Calcina, Cantore, Cerume, Cicuta, Collare, Coniato, Crinale, Cursore, Declino, Delfino, Devoto, Dimora, Ducato, Erede, Evaso, Fanale, Fastoso, Ferino, Forbito, Icona, Incroci, Infame, Irsuto, Ladrone, Legame, Lucroso, Lumino, Madrina, Matrice, Matrona, Minuta, Monsone, Moscato, Nativo, Nettuno, Odioso, Olivo, Ondoso, Opale, Ordito, Pagoda, Pedata, Peloso, Plotone, Puliti, Puzzone, Ramato, Rasone, Rettale, Rettore, Risata, Romito, Sapone, Scalfito, Scipito, Sentore, Siluro, Solare, Sottana, Sovrana, Tannino, Tellina, Tepore, Terrina, Tortino, Tossina, Uncino, Usura, Velina, Verdura, Vernice, Vorace.

High frequency words with non-dominant stress used in Experiment 3:

Abito, Alberi, Bergamo, Cagliari, Camera, Cinema, Comodo, Compito, Debito, Epoca, Esseri, Femmine, Fulmine, Genova, Giovane, Giudice, Indice, Lettera, Liberi, Limite, Macchina, Maschera, Merito, Nascita, Numeri, Nomina, Opera, Ordine, Ospite, Pagina, Perdita, Povera, Sabato, Semplice, Solito, Spirito, Svizzera, Termini, Vendita, Veneto, Vertice, Visita.

Low frequency words with non-dominant stress used in Experiment 3:

Afona, Alluce, Bigamo, Canfora, Carpino, Ceneri, Crimini, Concave, Decadi, Esodo, Ettari, Femore, Forbice, Gemito, Generi, Gettito, Inferi, Lamina, Lattice, Logoro, Macina, Mastice, Miseri, Nacchera, Nettare, Nomadi, Onice, Orbite, Orfane, Patina, Pecora, Proroga, Satura, Sagome, Scomoda, Segale, Sigari, Tartara, Vimini, Visceri, Viveri, Vortice.

Dominant stress word primes used in Experiment 4:

Ameno, Annoso, Arringa, Assolo, Atollo, Attinta, Avvezze, Bardato, Beffardi, Bislacco, Bivacchi, Bizzoso, Brandito, Brumosi, Calotte, Canuto, Cavillo, Combutta, Contrito, Dogale, Dovizia, Eczemi, Edotto, Effige, Facezie, Fachiri, Fervente, Franchigia, Frondose, Frugali, Gazzarra, Gendarmi, Gibboso, Gitano, Golpista, Ignavo, Incetta, Intonso, Invisa, Irriguo, Istrione, Lagnanza, Lapillo, Larvale, Loggiato, Ludibrio, Magione, Mercede, Miniati, Mozione, Nefande, Nequizia, Nuraghe, Oblunga, Ogiva, Olezzo, Oriundo, Orpello, Ossequi, Pasciuto, Pertugi, Presidio, Reietto, Retrivo, Rubizza, Satollo, Scafandro, Sevizie, Sollazzo, Stantia, Tarlato, Togati, Turgore, Uggioso, Uretra, Varato, Verdetti, Vessato, Vessillo, Vetuste, Vicari, Votivo, Zibibbo.

Non-dominant stress word primes used in Experiment 4:

Afona, Agave, Alluce, Andito, Angora, Argani, Argine, Augure, Bibita, Bifore, Bigamo, Bipede, Bisturi, Bosforo, Bretone, Canfora, Carpino, Celtica, Cerberi, Cernita, Concave, Crimine, Darsena, Decadi, Delega, Despota, Diafane, Dollaro, Edito, Egadi, Egloga, Esodo, Femore, Fiocine, Folaga, Folgore, Forfora, Fosforo, Fradice, Gamberi, Inclito, Indaco, Inferi, Inguine, Lamina, Lecito, Ligure, Logoro, Macina, Malaga, Mantice, Marmore, Onice, Orfani, Panama, Pomice, Postuma, Prodigo, Profuga, Proroga, Recluta, Redini, Remore, Rendita, Ricino, Ruderi, Satiro, Satura, Scomodo, Stipite, Suddita, Sudicie, Talamo, Talleri, Timpani, Tonaca, Tortora, Totani, Vortice, Zattera, Zefiro, Zigomo, Zingaro.

Nonword primes used in Experiment 4:

Abero, Adimo, Adoso, Aldume, Alima, Alona, Ammino, Antuce, Arfina, Ascimi, Astola, Astone, Bagica, Ballido, Batilo, Berice, Berino, Bildese, Birtona, Bistone, Bodulo, Bodune, Bollice, Botaro, Bovato, Bovero, Calave, Caleri, Calico, Calune, Canodo, Cavico, Cavola, Cecana, Celido, Cevema, Cevico, Ciovane, Codige, Cofera, Comilo, Compela, Condola, Corafo, Costice, Crimito, Delore, Defano, Denora, Dessile, Deturo, Dilaro, Dinuro, Disena, Efede, Efice, Ellate, Epede, Erale, Ertasi, Etaso, Fabato, Facero, Fagile, Faride, Fastole, Fecolo, Fedale, Fegore, Fessile, Fevole, Fevone, Forlata, Fosico, Frellata, Frotona, Fubito, Gamera, Gamito, Ganapa, Gatera, Gavere, Ginico, Gofano, Incroni, Impima, Iruta, Issubo, Iviero, Lagile, Lantiro, Legora, Lenolo, Licero, Lidame, Ligaro, Linata, Lisico, Mafona, Marcile, Mascato, Masole, Mavino, Mavida, Medulo, Mefolo, Merolo, Mevino, Mimore, Mosteri, Mubile, Necile, Oddimo, Olina, Onese, Ostore, Pabola, Paffica, Parico, Pavola, Parede, Paroga, Pidoso, Pifato, Pincolo, Pivida, Polaso, Polide, Povato, Privido, Prottola, Raddame, Regito, Retiro, Rifuta, Rivino, Rudomi, Sbaccole, Sentana, Sintura, Solume, Sorato, Stebore, Stofore, Stosimo, Subela, Tefala, Tivolo, Tegoro, Tentoro, Tevone, Tirloni, Tolebre, Tomuro, Trofulo, Tuposo, Vabore, Vemmina, Vernite, Vimbolo, Virpico, Zaloci, Zapide, Zelido, Zilaso, Zilota, Zolita
